# A genome draft of the legless anguid lizard, *Ophisaurus gracilis*

**DOI:** 10.1186/s13742-015-0056-7

**Published:** 2015-04-09

**Authors:** Bo Song, Shifeng Cheng, Yanbo Sun, Xiao Zhong, Jieqiong Jin, Rui Guan, Robert W Murphy, Jing Che, Yaping Zhang, Xin Liu

**Affiliations:** 1 BGI-Shenzhen, Shenzhen, 518083 China; 2 HKU-BGI Bioinformatics Algorithms and Core Technology Research Laboratory, The Computer Science Department, The University of Hong Kong, Hong Kong, China; 3 State Key Laboratory of Genetic Resources and Evolution, and Yunnan Laboratory of Molecular Biology of Domestic Animals, Kunming Institute of Zoology, Chinese Academy of Sciences, Kunming, 650223 China; 4 Centre for Biodiversity and Conservation Biology, Royal Ontario Museum, 100 Queen’s Park, Toronto, Ont. M5S 2C6 Canada; 5 Laboratory for Conservation and Utilization of Bio-resource, Yunnan University, Kunming, 650091 China

**Keywords:** Lizard genome, Anguidae, Squamate reptiles, Limblessness

## Abstract

**Background:**

Transition from a lizard-like to a snake-like body form is one of the most important transformations in reptilian evolution. The increasing number of sequenced reptilian genomes is enabling a deeper understanding of vertebrate evolution, although the genetic basis of the loss of limbs in reptiles remains enigmatic. Here we report genome sequencing, assembly, and annotation for the Asian glass lizard *Ophisaurus gracilis*, a limbless lizard species with an elongated snake-like body form. Addition of this species to the genome repository will provide an excellent resource for studying the genetic basis of limb loss and trunk elongation.

**Findings:**

*O. gracilis* genome sequencing using the Illumina HiSeq2000 platform resulted in 274.20 Gbp of raw data that was filtered and assembled to a final size of 1.78 Gbp, comprising 6,717 scaffolds with N50 = 1.27 Mbp. Based on the k-mer estimated genome size of 1.71 Gbp, the assembly appears to be nearly 100% complete. A total of 19,513 protein-coding genes were predicted, and 884.06 Mbp of repeat sequences (approximately half of the genome) were annotated. The draft genome of *O. gracilis* has similar characteristics to both lizard and snake genomes.

**Conclusions:**

We report the first genome of a lizard from the family Anguidae, *O. gracilis*. This supplements currently available genetic and genomic resources for amniote vertebrates, representing a major increase in comparative genome data available for squamate reptiles in particular.

## Data description

*Ophiosaurous gracilis* genomic DNA was extracted from the tail of a single male lizard collected from the Tibetan Plateau and used to construct seven paired-end Illumina libraries with insert sizes ranging from 180 bp to 20 kbp. To construct small-insert libraries (180, 500, and 800 bp), DNA was sheared to the target size range using Covair S2 (Covaris, Woburn, MA, USA) and ligated to adaptors. For long-insert libraries (2, 5, 10, and 20 kb), DNA was fragmented using a Hydroshear system (Digilab, Marlborough, MA, USA). Sheared fragments were biotin labelled at the ends and fragments of the desired size were gel purified. A second round of fragmentation was then conducted before adapter ligation. Both libraries were sequenced on an Illumina HiSeq2000 Genome Analyzer (Illumina, San Diego, CA, USA), with 100 bp and 90 bp sequencing for short insert size libraries (180–800 bp) and large insert size libraries (2–20 kbp), respectively. A total of 274.20 Gbp of raw data was generated, from which 147.08 Gbp of ‘clean’ data was obtained after removal of duplicates, contaminated reads (reads with adaptor sequences), low quality reads (with Solexa quality scores (Phred64) of less than 7 for >60% and >80% of bases for short-insert libraries and long-insert libraries, respectively) and reads with more than 10% ‘N’ bases. The *O. gracilis* genome size was estimated to be approximately 1.71 Gbp using a k-mer-based approach [1]. Based on this estimate, the clean data corresponds to approximately 86-fold coverage of the *O. gracilis* genome. High-quality reads were used for genome assembly (contig and scaffold construction) and gap closure was performed using the SOAPdenovo package and default parameters except that the k-mer size was set at 63 [2]. The final assembly had a total length of 1.78 Gbp, comprising 6,715 scaffolds assembled from 135,863 contigs, with the longest scaffold size being 6.68 Mbp. The N50 sizes for contigs and scaffolds were 23.41 kbp and 1.27 Mbp, respectively. Given the genome size estimate of 1.71 Gbp, genome coverage by the final assembly was probably complete, although this is probably a slight overestimate due to possible overlaps between some of the scaffolds and/or misassembly of some heterozygous alleles. Completeness of the assembly was confirmed by the successful mapping of up to 97% of reads from short insert libraries. Collectively, this data indicates that almost complete *O. gracilis* genome coverage was obtained.

Protein-coding genes were predicted and annotated by a combination of homology searching and *de novo* prediction using AUGUSTUS [3]. To search for homologous gene models, the genome assembly was queried against a database containing protein sequences and gene transcripts from three other squamate reptile species (*Anolis carolinesis*, *Ophiophagus hannah*, and *Python molurus bivittatus*) and four other tetrapod vertebrates (*Gallus gallus*, *Homo sapiens*, *Taeniopygia gutta*, and *Xenopus tropicalis*). This resulted in identification of a total of 19,513 protein-coding genes in the *O. gracilis* assembly, with an average of seven introns per gene. The gene length ranged from 137 to 96,389 bp, with an average of 1,506 bp; the average exon and intron length was 186 and 3,809 bp, respectively (Table 1).

**Table 1:** **Global statistics of the** ***O. gracilis*** **genome**

Statistic	Value
Size (Gb)	1.71
Scaffold number	6,715
Scaffold N50 (Mb)	1.27
Gene number	19,513
Average gene length (bp)	1,506
Average intron number	7
Average intron length (bp)	3,809
Average exon length (bp)	186

Genomic repeat elements in the *O. gracilis* genome assembly were also identified and annotated. RepeatMasker software version 3.2.7 [4] was used to search for repeat elements using the RepBase library (version 16.10) [5]. We also constructed a *de novo* repeat sequence database for the *O. gracilis* genome using LTR-FINDER [6] and RepeatModeler [7], and used this library to identify additional repeat elements using RepeatMasker. By combining the data obtained from both repeat element annotation approaches, a total length of 884.06 Mbp of the *O. gracilis* genome was identified as repetitive. Repeat annotations accounted for approximately 49.63% of the entire genome assembly, which is remarkably higher than estimates for other squamate reptiles, the anole lizard (~30.4%) [8] and both of the available snake genomes (the python (~27.60%) [9] and cobra (~31.28%) [10]). The repeat element landscape of *O. gracilis* mostly consists of retrotransposons, including long interspersed elements (LINEs), short interspersed elements (SINEs) and long terminal repeats (LTRs). LINEs represented the most abundant class of retrotransposons, occupying 37.65% of the genome, while the other repeat elements (SINE and LTR) comprised 1.80% and 6.44%, respectively (Table 2). DNA transposons were particularly rare, forming only 3.2% of the genome.

**Table 2:** **Summary of mobile element types**

Type	Length (kb)	Percentage of genome (%)
DNA	56,874	3.19
LINE	670,619	37.65
SINE	32,019	1.80
LTR	114,739	6.44
Other	177	0.01
Unknown	160,545	9.01
Total	884,057	49.63

In summary, we report the first annotated anguid lizard genome sequence assembly, to supplement the existing amniote genome resources in which squamate reptile sequences are sparsely represented. Despite the distant phylogenetic relationship [11], the morphology of the Asian glass lizard *O. gracilis* is highly convergent with that of snakes, including the lack of limbs and an elongated body. We therefore expect the genome of this species to be particularly useful for future comparative genomic analyses to identify the molecular basis of limb loss and body form evolution in squamate reptiles, and vertebrates in general.

## Availability of supporting data

Supporting data is available in the *GigaScience* repository, GigaDB [12], and raw data in the SRP052050.

## Abbreviations

EST: Expressed sequence tag
LINE: Long interspersed elements
LTR: Long terminal repeat
SINE: Short interspersed elements
